# rDNA Copy Number Variants Are Frequent Passenger Mutations in *Saccharomyces cerevisiae* Deletion Collections and *de Novo* Transformants

**DOI:** 10.1534/g3.116.030296

**Published:** 2016-07-22

**Authors:** Elizabeth X. Kwan, Xiaobin S. Wang, Haley M. Amemiya, Bonita J. Brewer, M. K. Raghuraman

**Affiliations:** * Department of Genome Sciences, University of Washington, Seattle, Washington 98195; † Department of Pathology and Cell Biology, College of Physicians and Surgeons, Columbia University, New York 10032

**Keywords:** rDNA, CNV, lithium, transformation, *Saccharomyces cerevisiae*

## Abstract

The *Saccharomyces cerevisiae* ribosomal DNA (rDNA) locus is known to exhibit greater instability relative to the rest of the genome. However, wild-type cells preferentially maintain a stable number of rDNA copies, suggesting underlying genetic control of the size of this locus. We performed a screen of a subset of the Yeast Knock-Out (YKO) single gene deletion collection to identify genetic regulators of this locus and to determine if rDNA copy number correlates with yeast replicative lifespan. While we found no correlation between replicative lifespan and rDNA size, we identified 64 candidate strains with significant rDNA copy number differences. However, in the process of validating candidate rDNA variants, we observed that independent isolates of our *de novo* gene deletion strains had unsolicited but significant changes in rDNA copy number. Moreover, we were not able to recapitulate rDNA phenotypes from the YKO yeast deletion collection. Instead, we found that the standard lithium acetate transformation protocol is a significant source of rDNA copy number variation, with lithium acetate exposure being the treatment causing variable rDNA copy number events after transformation. As the effects of variable rDNA copy number are being increasingly reported, our finding that rDNA is affected by lithium acetate exposure suggested that rDNA copy number variants may be influential passenger mutations in standard strain construction in *S. cerevisiae*.

The genes encoding the RNA components of ribosomes, or ribosomal DNA (rDNA), are present as highly repetitive and variable elements in many eukaryotic genomes (Prokopowich *et al.* 2003). Human rDNA copy number varies widely between individuals and has been reported to range from 14 copies to 400 copies (Gibbons *et al.* 2015). Copy number variation of rDNA sequences has also been documented between individuals of mice, flies, and yeast, as well as between different species and different strains within a species (Gibbons *et al.* 2015; James *et al.* 2009; Paredes *et al.* 2011; Pasero and Marilley 1993; Stults *et al.* 2008). In the budding yeast *Saccharomyces cerevisiae*, rDNA copy number is known to be plastic yet the wild-type laboratory strain preferentially returns to ∼150 rDNA repeats after copy number perturbation (Kobayashi *et al.* 1998), suggesting that there is internal regulation of rDNA array size. Several regulators of *S. cerevisiae* rDNA copy number have in fact been identified. Known regulators include the rDNA itself (Kwan *et al.* 2013), RNA polymerase I (Rpa135) (Brewer *et al.* 1992), components of the origin recognition complex (ORC; *orc1-4* and *orc2-1*) (Ide *et al.* 2007), and the DNA damage-responsive S-phase histone acetyltransferase Rtt109 (Ide *et al.* 2013).

Consistent with the idea of homeostatic maintenance of rDNA copy number, mounting evidence has indicated that rDNA copy number has functional repercussions on different cellular processes. Michel *et al.* (2005) have described that a 30–50% reduction of rDNA repeats can enhance chromatin silencing at telomeric and mating type loci in the budding yeast *S. cerevisiae*, suggesting that rDNA can have long range effects on overall gene expression. This observation was further supported by recent work by Paredes *et al.* (2011) and Gibbons *et al.* (2015), who have linked rDNA copy number variation in *Drosophila melanogaster* and humans to gene expression changes, primarily in metabolic and mitochondrial pathways. Reductions in rDNA copy number have also been associated with beneficial responses to DNA replication stress and yeast longevity (Ide *et al.* 2007; Kwan *et al.* 2013). Additionally, there has been longstanding interest in the association between rDNA locus instability and yeast replicative age (Ganley *et al.* 2009; Lindstrom *et al.* 2011; Sinclair *et al.* 1997; Sinclair and Guarente 1997). Any novel regulators of rDNA copy number could therefore have significant indirect associations with gene expression, chromatin regulation, DNA replication, aging, and other cellular functions.

Here, we outline our efforts at both identifying novel regulators of *S. cerevisiae* rDNA copy number and investigating the potential relationship between rDNA copy number and yeast longevity. Our screen of 221 long-lived mutants and 213 nonlong-lived strains from the Yeast Knock-Out (YKO) collection reveals significant rDNA copy number variation in the mutant strains; however, we could find no correlation between longevity and rDNA copy number. Further examination of candidate YKO strains with significantly altered rDNA copy number reveals that the identified gene deletions are not responsible for the changes to rDNA array size. Instead, we find that standard lithium acetate transformation protocols can result in seemingly random but significant rDNA copy number changes in a proportion of recovered colonies, regardless of whether selectable transforming DNA was present. Our findings suggest that rDNA copy number regulators may be affected by lithium acetate and that rDNA copy number variants (CNVs) may be influential passenger mutations generated during standard strain construction protocols.

## Materials and Methods

### Yeast strains and media

The rDNA copy number screen was performed with strains from the *MAT*α YKO single gene deletion collection generated by the *Saccharomyces* Gene Deletion Project (Winzeler *et al.* 1999, http://www-sequence.stanford.edu/group/yeast_deletion_project/deletions3.html) and obtained from OpenBiosystems. From McCormick *et al.* (2015), we obtained a list of 221 single gene deletions identified in their screen of the *MATa* and *MAT*α YKO collections as extending replicative lifespan in the S288c background. We picked 213 control strains from microtiter wells horizontally adjacent to long-lived strains. Each YKO strain was grown on YPD plates, inoculated into YPD medium (2% glucose), grown to stationary phase at 30°, and collected for DNA preparation in agarose plugs. For *de novo* gene deletions and transformation assays we used the *MATa* S288c strain BY4741 and also a *MATa* strain in the A364a background (BB14-3a; McCune *et al.* 2008). For the initial screen of the Magic Marker deletion collection (Pan *et al.* 2004; Tong *et al.* 2001), strains were inoculated directly from the microtiter library plates into new microtiter plates with 200 µl YPD medium and then grown to stationary phase at 30°. For single colony experiments, Magic Marker strains were grown on YPD plates, inoculated into YPD medium (2% glucose), and grown to stationary phase at 30°.

Strains are available upon request. Supplemental Material, Table S1 contains the primer sequences for the gene replacement fragments used for *de novo* gene deletion. Table S2 contains the list of *MAT*α YKO strains examined and the rDNA copy number estimate for each strain.

### Preparation of genomic DNA in agarose plugs

Genomic DNA was prepared using a modified protocol obtained from Dr. Juan Lucas Argueso (personal communication), in which approximately 2 × 10^8^ cells (1 ml) from each stationary phase culture were embedded in agarose plugs. Cells were resuspended in 180 µl of 0.5% low-melt SeaPlaque agarose (in 100 mM EDTA) with 12 µl of 25 mg/ml Zymolyase 20-T (Amsbio). The agarose mixture was then pipetted into two Bio-Rad plug molds to solidify at 4° for 30 min. Plugs were extruded from the plug mold into 24-well plates, two plugs per well, and incubated in 2 ml of 500 mM EDTA + 10 mM Tris at 37° overnight. 400 µl of 5 mg/ml proteinase K (Roche) in 500 mM EDTA were then added into each well and incubated at 50° for 5 hr. Plugs were subjected to four 1 hr washes with TE (10 mM Tris, 1 mM EDTA, pH 8.0) before being stored in TE at 4°. For restriction enzyme digests, plugs were washed eight times with TE before two 30 min washes in restriction buffer + BSA.

### Measurement of chromosome XII size and estimation of rDNA copy number

We used CHEF (contour-clamped homogeneous electric field) gel electrophoresis to measure the size of chromosome XII, the chromosome that contains the rDNA locus. A slice of agarose plug was embedded in a 1% agarose gel (0.5 × TBE) along with a wild-type sample as reference. We performed the electrophoresis in 2.3 L of 0.5 × TBE using a Bio-Rad CHEF electrophoresis cell at 100 V for 68 hr, switch time ramped from 300 to 900 sec (Kobayashi *et al.* 1998). The gel was then stained with ethidium bromide to visualize all chromosomes. To examine the size of the excised rDNA array, genomic DNA samples in plugs were digested with *Bam*HI and then separated by CHEF gel electrophoresis on a 1% agarose gel at 165 V for 24 hr (switch time = 40–170 sec).

**Figure 1:**
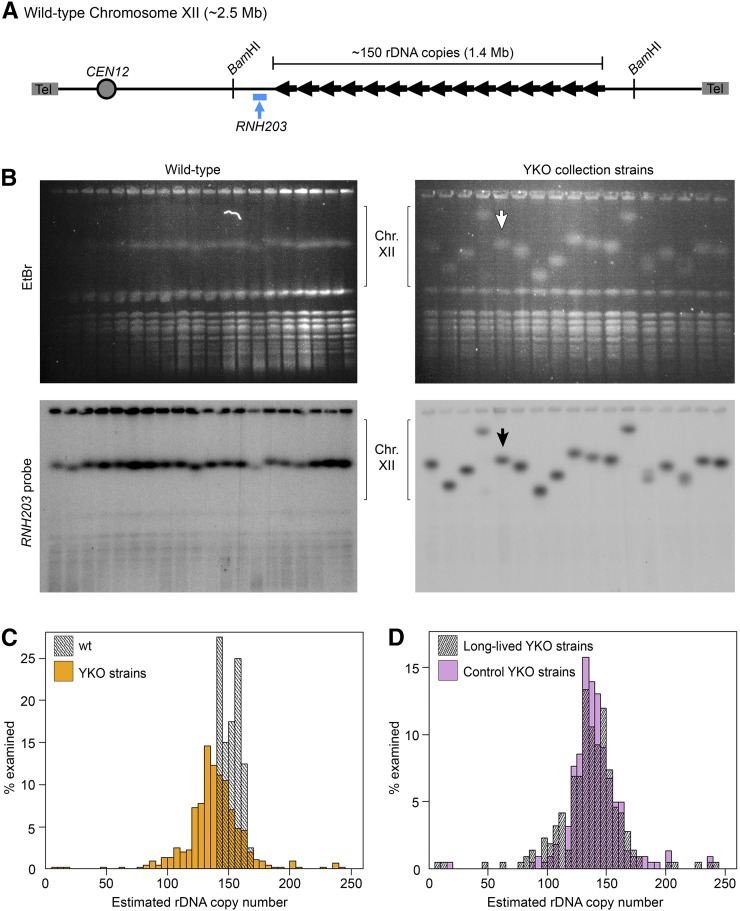
Detection of rDNA copy number variation between wild-type clones and YKO deletion strains. (A) The 150 repeats of the wild-type *S. cerevisiae* rDNA locus comprise ∼1.4 Mb of Chromosome XII. Relative locations of the rDNA-flanking *Bam*HI sites and single copy *RNH203* probe sequence used for this study are as indicated. (B) Top: ethidium bromide-stained CHEF gels of 20 independent wild-type clones and 16 YKO strains (wild-type control indicated by arrow) shows positions of all 16 *S. cerevisiae* chromosomes, with chromosome XII generally being the largest. Bottom: Southern blots of the same gels hybridized with an *RNH203* probe, a single copy gene on chromosome XII. (C) rDNA copy number distribution of 40 wild-type clones and the 434 YKO collection strains examined by CHEF gel analysis (Wilcoxon rank-sum: *P* < 0.0001). (D) rDNA copy number distribution of long-lived YKO strains and control YKO strains (Wilcoxon rank-sum: *P* = 0.196). CHEF, contour-clamped homogenous electric field; Chr, chromosome; EtBr, ethidium bromide; rDNA, ribosomal DNA; Tel, telomere; wt, wild-type; YKO, Yeast Knock-Out.

Chromosome XII size and rDNA copy number were further examined via Southern blotting. DNA from each CHEF gel was transferred to a GeneScreen Hybridization membrane using standard Southern blotting protocols. We then hybridized using a ^32^P-labeled probe for *RNH203*, a single copy gene located on chromosome XII just centromere-proximal to the rDNA region and within the *Bam*HI-restriction fragment (Figure 1A). The blots were exposed to X-ray film and to Bio-Rad Molecular Imaging FX phosphor screens for visualization and quantification of signal intensity. Phosphor screens were scanned using a Bio-Rad Personal Molecular Imaging scanner and analyzed using Bio-Rad’s Quantity One software. For each gel, we used the Quantity One program to generate a standard curve from wild-type chromosome sizes to estimate chromosome XII size for every YKO strain, from which we calculated the approximate endogenous rDNA copy number. To calculate rDNA copy number using the size of the excised rDNA locus, 39.7 kb was subtracted from the size of the band to account for the nearest external *Bam*HI sites (8.8 kb and 30.9 kb), then divided by the 9.1 kb rDNA repeat size. A Wilcoxon Rank-Sum test (KaleidaGraph software) was used to assess significance of the differences between wild-type and YKO strains, and also between long-lived and control deletion strains. To determine correlation between our data and data from Saka *et al.* (2016), we used R software to calculate Pearson correlations.

Relative migration distance measurements for chromosomes XII, IV, and VII were obtained by measuring the distance (*d*) from the well to each respective chromosome. For each sample, the relative distance was calculated by dividing its chromosome XII measurement by its chromosome VII measurement (*d*_(XII)_ / *d*_(VII)_). This calculation was similarly repeated for chromosome IV (*d*_(IV)_ / *d*_(VII)_) as a control. Migration difference from wild-type for each sample was calculated by subtracting the untreated wild-type value (sample – wt). Significance of chromosome XII size variation between cohorts of strains was assessed using a Wilcoxon Rank-Sum test of the absolute value of migration difference for each sample (|sample – wt|) using KaleidaGraph software. For comparison with the 40 wild-type, *de novo*, and mock transformation isolates, a comparably sized population of 40 YKO strains was selected from each of the 15 YKO screen gels by rolling a 20-sided die.

### Yeast lithium acetate transformation and de novo gene deletion

For *de novo* gene deletion, we used a modified transformation protocol from the *Saccharomyces* Gene Deletion Project (http://www-sequence.stanford.edu/group/yeast_deletion_project/transprot.html), similar to the protocol by Gietz *et al.* (1992). We designed primers for gene replacement with either *KanMX* or *URA3* (Table S1). DNA fragments for *de novo* gene deletion were PCR amplified from a plasmid (pRS400-KanMX6, pRS305, or pRS306), ethanol precipitated, and resuspended in 20 µl TE. Wild-type BY4741 cells were grown in YPD overnight and then diluted to an OD_660_ = 0.2 in 50 ml fresh YPD. Cells were allowed to grow approximately two doublings to OD_660_ = 0.7 before pelleting, washing in 20 ml TE, washing in 20 ml TE-LiAc (10 mM Tris-HCl pH 8.0, 1 mM EDTA pH 8.0, and 100 mM lithium acetate), and resuspending in 700 µl of TE-LiAc. For each transformation, 20 µl boiled 8 mg/ml herring testes DNA (ssDNA carrier), 1 ml PEG-TE-LiAc (40% PEG-4000, 10 mM Tris-HCl pH 8.0, 1 mM EDTA, and 100 mM lithium acetate), and transforming DNA were added to 100 µl of competent cells. Cells were then incubated at 30° for 30 min and subjected to heat shock at 42° for 30 min. Cells were then plated on either drop-out plates for auxotrophic marker selection or onto YPD for replica plating onto YPD+G418 the following day for KanMX selection. *De novo* deletion strains were checked by PCR and then Southern blotting. Mock transformations were performed as above without selectable transforming DNA and plated on nonselective YPD plates.

### Yeast transformation by spheroplasting

The spheroplast transformation protocol was adapted from Hinnen *et al.* (1978). A single BY4741 colony was inoculated into 5 ml YPD and grown overnight. In the morning, cells were diluted in fresh YPD to a density of 4 × 10^6^ cells/ml and were grown to a density of 4 × 10^7^ cell/ml. At this point, a small amount of culture was then plated for single colonies for controls. Next, 1.5 × 10^9^ cells were collected, washed once with 20 ml water, washed once with 20 ml 1 M sorbitol, and resuspended in 20 ml SCEM (1 M sorbitol, 0.1 M sodium citrate, and 10 mM EDTA) with 43 µl β-mercaptoethanol and 60 µl of 25 mg/ml Zymolyase 20-T (Amsbio). Cells were incubated at 30° until 90% spheroplasted (15 min) and then gently pelleted at 240 × *g* for 5 min, washed twice with 20 ml STC (1 M sorbitol, 10 mM Tris-HCl pH 7.5, and 10 mM CaCl_2_), and resuspended in 2 ml STC. For transformation, 1 × 10^8^ spheroplasts (140 µl) were transferred to a 1.5 ml microfuge tube with or without 100 ng of pRS426 and incubated at room temperature for 10 min. One milliliter of PEG solution (10 mM Tris-HCl pH 7.5, 10 mM CaCl_2_, and 20% PEG-8000) was then added and incubated for another 10 min at room temperature. Spheroplasts were then pelleted at 300 × *g*, resuspended in 200 µl SOS (1 M sorbitol, 6.5 mM CaCl_2_, 0.25% yeast extract, and 0.5% bactopeptone), and incubated at 30° for 30 min. To plate, 40 µl of cells were mixed with 200 µl SOS and 8 ml top agar [for 500 ml: 91 g sorbitol, 3.4 g yeast nitrogen base with ammonium sulfate, 12.5 g agar, 2% glucose, and 0.39 g of CSM-ura powder (Sunrise Science)] and spread immediately onto YPD or C-URA sorbitol plates [for 500 ml: 91 g sorbitol, 3.4 g yeast nitrogen base with ammonium sulfate, 10 g agar, 2% glucose, and 0.39 g of CSM-ura powder (Sunrise Science)]. Plates were incubated at 30° for 3 d and transformant isolates were then streaked out for single colonies.

### Yeast transformation by electroporation

The same culture used for the spheroplasting protocol above was used for electroporation, except that the culture was grown to a density of 8 × 10^7^ cell/ml. 50 ml of cells were pelleted, washed once with 40 ml ice-cold water, once with 20 ml ice-cold water, and once with 5 ml of 1 M sorbitol. Cells were resuspended in 150 µl of 1 M sorbitol and kept on ice. For transformation, 40 µl of cell suspension and 100 ng pRS426 were mixed in a prechilled 0.2 cm electroporation cuvette and then electroporated using a Bio-Rad MicroPulser on the Fungi/Sc2 setting (1.5 kV, 25 uF, and 200 Ohms). One milliliter of 1 M sorbitol was added immediately after electroporation and cells were plated on selective plates (200 µl/plate, C-URA or YPD plates). After incubation at 30° for 2 d, transformant isolates were streaked out for single colonies.

### Data availability

The authors state that all data necessary for confirming the conclusions presented in the article are represented fully within the article.

## Results

### Screen of yeast deletion collection strains for altered rDNA copy number

We had two main interests in performing a screen for strains with altered rDNA copy number. First, we were interested in identifying genes required for maintaining the number of repeats in the wild-type rDNA array. Second, we were interested in determining if there was a relationship between rDNA copy number and replicative lifespan. To address both of these interests in a concerted manner, we assessed rDNA copy number in 434 *MAT*α single gene deletion YKO strains: 221 long-lived YKO strains reported by McCormick *et al.* (2015) along with a control population of 213 control strains from the same YKO collection. The strains in the control YKO group had been examined by McCormick *et al.* (2015) but were reported as not having increased longevity. The rDNA copy number of each strain was estimated based on the size of chromosome XII from the CHEF gel (Figure 1A and Table S2). The accuracy of our rDNA copy number estimation was confirmed by the strong correlation between those estimates and the size of the *Bam*HI-excised rDNA array in several YKO strains (R^2^ = 0.968, Figure S1). We also surveyed 40 wild-type single colonies to assess the range of natural rDNA copy number variation. We found that there is minor variation between wild-type colonies, but observed that many strains in the YKO collection have dramatically different rDNA copy numbers (Figure 1B). The distribution of rDNA copy number found in the YKO collection follows an almost normal distribution (mean = 139 ± 25), which is broader than the natural variation seen among clones of the wild-type strain (mean = 152 ± 7, Figure 1C) and is significantly different from the distribution of rDNA copy number seen in the wild-type clones (*P* < 0.0001).

### Long-lived YKO strains do not have a significant rDNA copy number bias

To assess whether rDNA copy number is linked to yeast replicative lifespan, we examined the rDNA copy number between long-lived YKO strains and those from the YKO control group. Based on the findings by Kwan *et al.* (2013), we predicted that having a smaller rDNA array would directly promote yeast longevity, and therefore long-lived strains would have fewer rDNA copies than the control strains. However, examination of rDNA copy number distributions reveals almost complete overlap between long-lived YKO strains (mean = 141 ± 22) and control YKO strains (mean = 137 ± 27) and no significant difference in rDNA copy number distinguishes long-lived strains (Figure 1D, *P* = 0.196). We therefore conclude that there is no direct relationship in the YKO collection strains between replicative lifespan and the number of rDNA repeats.

### rDNA copy number variation seen in YKO collection is not due to the deleted gene

**Figure 2:**
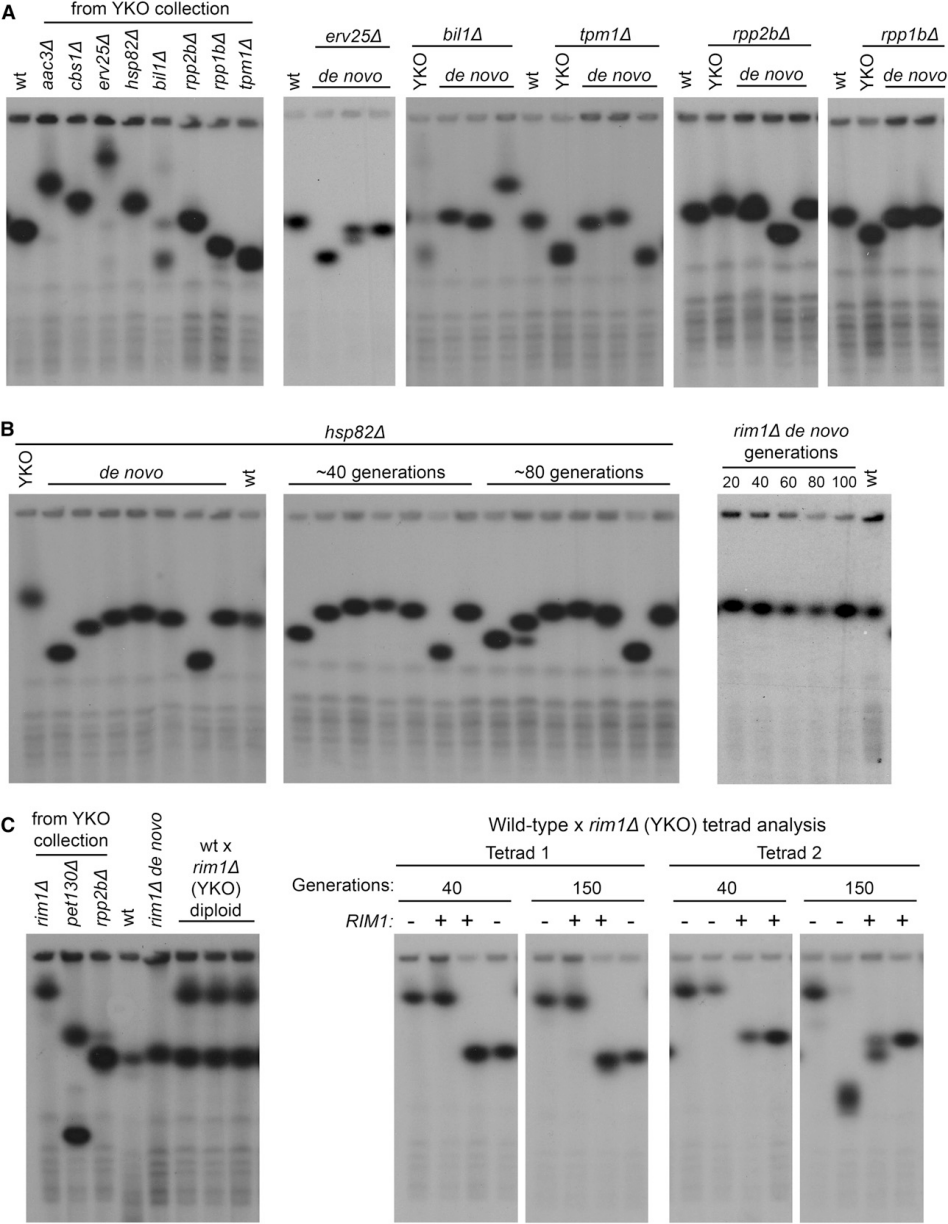
Assessing the causality of YKO mutations on rDNA copy number phenotypes: *de novo* gene deletion, serial passaging, and meiotic segregation. Southern blots of CHEF gels probed with *RNH203* (chromosome XII). Wild-type and original YKO strain samples were included for comparison. (A) Left panel: chromosome XII sizes of chosen candidate strains taken directly from the YKO collection. Right panels: Southern blots showing chromosome XII sizes of multiple isolates from each *de novo* gene deletion: *erv25*Δ, *bil1*Δ, *tpm1*Δ, *rpp1b*Δ, and *rpp2b*Δ. (B) *De novo* gene deletion strains of *hsp82*Δ and *rim1*Δ were examined by CHEF gel electrophoresis over the course of 80–100 generations of growth. (C) Left panel: CHEF gel examining chromosome XII in the *rim1*Δ strain from the *MAT*α YKO collection, wild-type *MATa* strain BY4741, and the resulting *rim1*Δ*/RIM1* heterozygous diploid strain. Right panels: chromosome XII sizes of *rim1*Δ and *RIM1* haploid strains generated from diploid sporulation examined after 40 and 150 generations. rDNA copy number randomly segregated in both *rim1*Δ and *RIM1* spores and the inherited rDNA copy number was maintained after 150 generations in all but one *rim1*Δ strain, which reduced rDNA copy number. CHEF, contour-clamped homogenous electric field; rDNA, ribosomal DNA; wt, wild-type; YKO, Yeast Knock-Out.

We identified 61 YKO strains with rDNA arrays that were longer or shorter by at least one standard deviation (≤ 115 copies or ≥ 180 copies) and chose to verify the effects of several candidate genes on rDNA copy number: *AAC3*, *BIL1*, *CBS1*, *ERV25*, *HSP82*, *PET130*, *RIM1*, *RPP1B*, and *TPM1* (Figure 2, A and C). We included *RPP2B* as a control since the YKO *rpp2b*Δ strain was identified as having a wild-type rDNA number. These nine strains were checked for the correct deleted gene and all had the correct *KanMX* sequence except the strains annotated as *aac3*Δ and *cbs1*Δ. These two strains had wild-type versions of their respective genes and were therefore excluded. We then generated separate *de novo* deletions of the seven remaining genes in the list above in wild-type strain BY4741 using lithium acetate transformation, verified gene deletion by PCR and Southern blotting, and assessed at least three independent transformants for rDNA copy number using CHEF gel electrophoresis/Southern blotting (Figure 2A). Surprisingly, we found that the majority of *de novo* gene deletion isolates maintained their wild-type rDNA array size (24/34 isolates). The rest of the transformants had seemingly random changes to rDNA size, often trending in the opposite direction to the phenotype seen in the original YKO strain. Even *rpp2b*Δ, our YKO candidate with a wild-type rDNA copy number, produced *de novo* deletion isolates with altered rDNA size. The inconsistent rDNA copy number changes suggested that rDNA changes observed in the *de novo* gene deletion isolates were not likely to be due to the loss of the deleted genes.

A previous study showed that the rDNA array requires 120 generations to recover from a reduction down to two copies back to 150 copies (Kobayashi *et al.* 1998). Given the lack of concordance in rDNA copy numbers between the YKO deletions and our *de novo* deletions, we wondered if the rDNA array required the passage of multiple generations to reach copy number equilibrium in *de novo* gene deletion strains. Accordingly, we serially passaged *de novo* isolates of *hsp82*Δ and *rim1*Δ on plates for 80–100 generations. Despite this long period of growth, we found that the rDNA copy number in each isolate was stably maintained (Figure 2B). The one change we did observe was that an isolate of *hsp82*Δ accumulated a subpopulation of cells with fewer rDNA repeats, which is opposite to the increased rDNA copy number seen in the YKO *hsp82*Δ strain. These results indicate that the rDNA arrays in different isolates are stable and that passaging does not lead to the new equilibrium at the copy number seen in the YKO deletion strains.

As an independent test of whether the rDNA copy number phenotype in a YKO strain is causally associated with the gene deletion, we crossed the wild-type strain BY4741 (150 rDNA copies) to the YKO *rim1*Δ strain (*MAT*α, 237 rDNA copies) and sporulated the resulting *rim1*Δ/*RIM1* heterozygous diploid strain for tetrad analysis. If the increased rDNA copy number were the consequence of the *RIM1* deletion, then regardless of the rDNA copy number initially inherited by *rim1*Δ spores, the rDNA array in those spore clones would attain the larger size after serial passaging. If the two phenotypes were unrelated, then the different chromosome XII sizes would segregate randomly in the tetrads and would not show any association with the *RIM1* genotype even after serial passaging. We observed that the parental wild-type and *rim1*Δ rDNA array sizes are maintained in the diploid strains and segregate randomly in the resulting tetrad spores. In keeping with the latter possibility outlined above, even after 40 and 150 generations of growth (Figure 2C), we did not see any changes that associate *rim1*Δ with the increased rDNA copy number seen in the YKO collection strain. In fact, one *rim1*Δ isolate that had inherited the larger rDNA locus had drastically reduced its rDNA copy number after 150 generations. Therefore, we conclude that the deleted genes in candidate YKO strains are not generally responsible for the changes to rDNA copy number observed in those strains.

A recently published rDNA screen by Saka *et al.* (2016) examined all 4876 strains in the *MATa* YKO deletion collection and ranked each strain into four rDNA size classes: s-Class 1 (< 80 copies), s-Class 2 (wild-type copy number), s-Class 3 (200–450 copies), and s-Class 4 (> 450 copies). We compared the rDNA copy number estimates from our YKO screen with those from Saka *et al.* (2016), but found no concordance between our rDNA copy number estimates and their size classifications (R = 0.039, *P* = 0.413, Figure S2A). For example, we identified the *MAT*α YKO *rim1*Δ strain as having 237 rDNA copies, but in the screen by Saka *et al.* (2016), the *MATa* YKO *rim1*Δ strain had the wild-type rDNA copy number (150 copies). It is possible that rDNA regulation is different between the *MATa* and *MAT*α YKO collections, which would require hundreds of mating-type specific genetic interactions to account for the widespread disparity in rDNA phenotypes. To examine this possibility, we attempted to use the *MATa* S288c strain BY4741 to reproduce the increased rDNA copy number phenotype seen in an *rtt109*Δ mutant (Ide *et al.* 2013), the only verified mutant from the 239 YKO rDNA copy number mutants identified by Saka *et al.* (2016). Our *de novo rtt109*Δ transformants did not recapitulate the increased rDNA copy number seen by Ide *et al.* (2013) (Figure S2B). This discrepancy in *de novo rtt109*Δ rDNA phenotypes could be due to the strain differences between the S288c strains used for the YKO collections and the W303 strain used for *de novo* gene deletion in their study. The lack of correlation between two screens of the YKO deletion collection further underscored the disconnect we found between the rDNA copy number phenotypes and the gene deletions in the YKO strains.

### Magic Marker single gene deletion strains often have heterogeneous chromosome XII sizes

**Figure 3:**
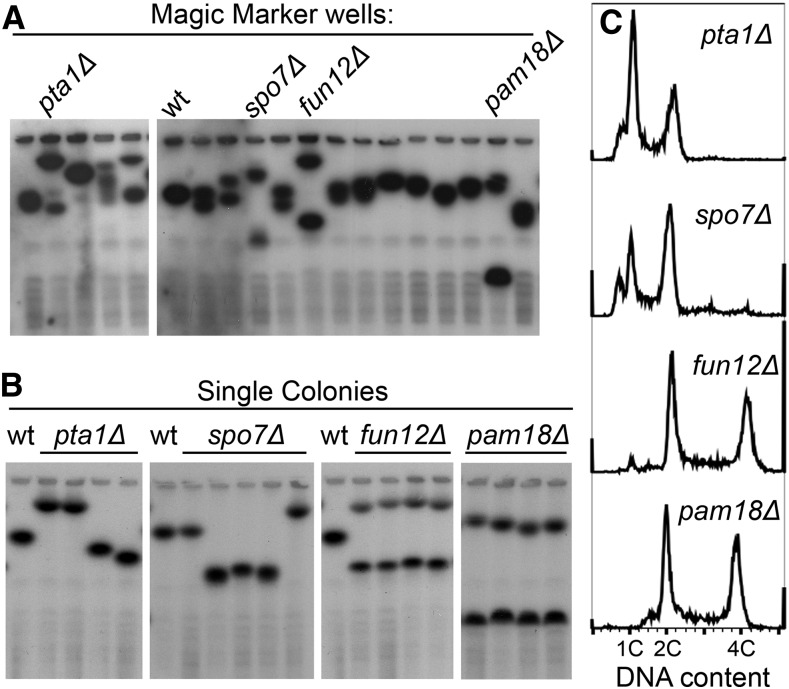
The Magic Marker haploid deletion collection exhibits rDNA copy number heterogeneity. (A) Strains from the Magic Marker collection were inoculated directly from the library wells into 96-well plates, grown overnight, and collected for CHEF gel and Southern blot analysis. Multiple chromosome XII bands were present in many Magic Marker collection strains such as *pta1*Δ, *spo7*Δ, *fun12*Δ, and *pam18*Δ. (B) Single colonies were isolated from Magic Marker strains for CHEF gel analysis. Each colony from strains *pta1*Δ or *spo7*Δ possessed a single chromosome XII band, whereas *fun12*Δ or *pam18*Δ colonies each maintained more than one chromosome XII band. (C) Flow cytometry analysis of strains from the Magic Marker collection cultures indicate that the *pta1*Δ and *spo7*Δ strains were haploid while *fun12*Δ and *pam18*Δ were diploid. CHEF, contour-clamped homogenous electric field; rDNA, ribosomal DNA; wt, wild-type.

To determine whether rDNA size variation was unique to the YKO collection, we characterized rDNA copy number heterogeneity in another yeast deletion library, the Magic Marker collection. The haploid Magic Marker/SGA reporter collection was generated from the sporulation of heterozygous deletion diploid strains, avoiding selection for compensatory mutations seen in haploid and homozygous gene deletion collections (Pan *et al.* 2004; Tong *et al.* 2001). We examined approximately 200 strains from the Magic Marker collection and found not only a significant amount of rDNA heterogeneity between mutants but, more surprisingly, more than one distinct chromosome XII band within a single strain (Figure 3A). For each strain that had two or more rDNA bands, we examined single colonies by barcode sequencing, CHEF gel electrophoresis, and flow cytometry. All single colonies from each strain had the correct barcode sequence and we therefore rejected the possibility that the multiple chromosome XII bands were results of cross contamination between wells in the microtiter dishes. Single colonies that retained only one chromosome XII size were verified as haploid with different isolates from the same strain having different chromosome XII sizes (Figure 3, B and C). This finding suggested that haploid cultures taken from library wells contained a mixed population of cells with different chromosome XII sizes and reflects our tetrad analysis results with the faithful inheritance of parental rDNA copy numbers in the resulting spores. Single colonies that retained two chromosome XII bands were found by flow cytometry to be diploid strains (Figure 3, B and C) that were heterozygous for rDNA CNVs.

### Lithium acetate transformation contributes to rDNA copy number changes

**Figure 4:**
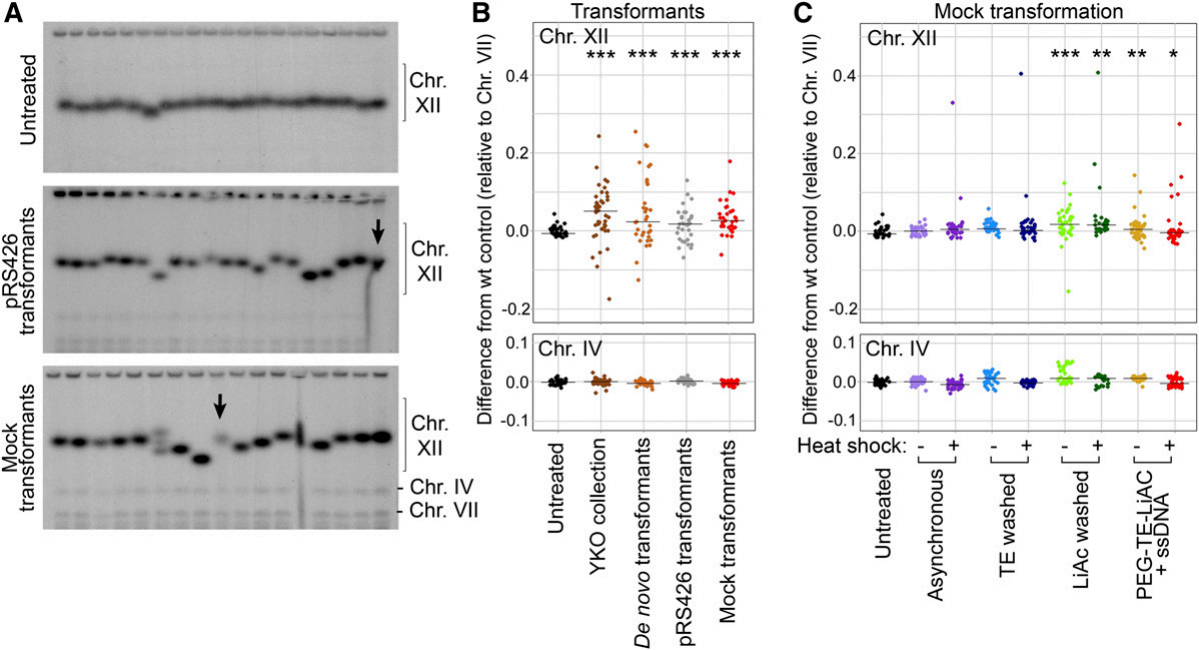
rDNA copy number variants are generated during transformation after lithium acetate exposure. (A) CHEF gel electrophoresis of 19 single colonies isolated from an untreated asynchronous culture, after transformation with the plasmid pRS426, or after mock transformation without selectable DNA. A common untreated control sample was run on each gel (arrows). (B) The plots show chromosomes XII and IV variation in wild-type untreated control isolates, YKO collection strains, *de novo* gene deletion transformants, plasmid pRS426 transformants, and mock transformants (*** *P* < 0.0001 in comparison to the untreated wild-type controls). The distribution of chromosome IV sizes indicated that there was little variation in this chromosome between isolates (plotted on the same scale). (C) The plots show chromosomes XII and IV variation in isolates from different stages of a mock transformation protocol. Each transformation group was compared to untreated controls [Wilcoxon Rank-Sum: *P* < 0.0001 (***), < 0.01 (**), < 0.05 (*)]. CHEF, contour-clamped homogenous electric field; Chr., chromosome; LiAc, lithium acetate; PEG, polyethylene glycol; rDNA, ribosomal DNA; ssDNA, single-stranded DNA; TE, Tris-EDTA; wt, wild-type; YKO, Yeast Knock-Out.

What is the source of rDNA copy number variation found in the YKO collection if not the particular genes being deleted? We found little variation in the chromosomal rDNA locus size across the 40 wild-type colonies we examined, suggesting that rDNA copy number is stable under normal conditions, yet 14% of the YKO strains we surveyed (61 of 434) had a significantly altered rDNA copy number. Additionally, approximately 30% of our *de novo* deletion strains had unsolicited and inconsistent rDNA copy number variation. The variation seen in both of these groups is significantly larger than the variation in untreated wild-type colonies (*P* < 0.0001, Figure 4B), suggesting that a hitherto hidden variable in the generation of genetically altered yeast strains could be affecting the chromosomal rDNA locus; specifically, the process of transformation itself could be contributing to rDNA copy number changes.

One commonality between the creation of the YKO deletion strains and our *de novo* deletion strains was transformation and subsequent selection for the incorporated marker. We therefore first considered the possibility that transformation and the bottleneck produced by selection for the selectable marker was stimulating or selecting for rDNA instability. To test this possibility, we transformed wild-type cells with the nonintegrating, circular plasmid pRS426. Among 35 transformed isolates that we tested (Figure 4A), we observed greater variation in chromosome XII size than seen in untreated wild-type cells (*P* < 0.0001, Figure 4B). We therefore concluded that cells acquire chromosomal rDNA copy number changes as a result of transformation.

To test whether the bottle neck produced by selecting for transforming DNA is necessary for the copy number changes, we examined chromosome XII in isolates from our control samples that had been taken through mock lithium acetate (LiAc) transformation without plasmid. We were astonished to find heterogeneous rDNA copy numbers in cells taken through mock transformation (*P* < 0.001, Figure 4, A and B), suggesting that the transformation process itself results in rDNA copy number variation. This response was not specific to the S288c strain background, as we found similar—or perhaps even greater—rDNA size heterogeneity after mock transformation of a different laboratory strain, BB14-3a (A364a background) (Figure S3). These results suggested that some aspect of the LiAc transformation procedure itself could be inducing changes to the chromosomal rDNA locus in *S. cerevisiae*.

### Exposure to lithium acetate results in increases in rDNA CNVs

To identify the transformation step(s) that were inducing rDNA copy number changes, we performed mock LiAc transformation and analyzed chromosome XII size from single colonies obtained after each transformation step. We found that rDNA size was stable in cells from asynchronous cultures and from cells that were washed with TE, but cells exposed to LiAc produced a number of isolates with significant rDNA copy number variation (Figure 4C and Figure S4). We also observed some rDNA CNVs from cells taken through heat shock alone, though not enough to be statistically significant. The observation that rDNA CNVs were observed in all treatments with LiAc suggests the presence of a mechanism that mediates cellular responses to LiAc stress and interferes with rDNA array maintenance.

Finally, we tested whether alternative transformation procedures—such as electroporation or spheroplasting (Kawai *et al.* 2010)—also lead to rDNA CNV. We subjected cells from a common BY4741 culture to either electroporation or spheroplast transformation, with or without selectable DNA (pRS426), and analyzed 38 isolates from each treatment via CHEF gel and Southern blotting (only 19 pRS426 spheroplast transformants were examined due to low transformation efficiency). While 5% of isolates taken through either LiAc-free transformation protocol exhibited mild rDNA copy number fluctuations, no significant difference was found between untreated cells and cells taken through electroporation/spheroplast transformation (Figure S5, *P* > 0.2). We therefore conclude that transformation itself is not a general source of rDNA CNVs.

## Discussion

We present this cautionary missive regarding the highly plastic *S. cerevisiae* rDNA locus in genome-wide collections and in response to standard genetic manipulation protocols as a public service announcement for prospective future analyses using transgenic strains. Our screen for mutants with altered rDNA copy numbers appeared to identify many candidates from the YKO deletion collection. Unfortunately, we determined that the relevant gene deletions are not responsible for the significant changes to rDNA copy number seen in our candidate strains. Our results instead suggest that the rDNA copy number variants stem from events occurring during standard transformation protocols using lithium acetate. We find that these rDNA copy number alterations are stable, occur after exposure to lithium acetate, and are produced independently of selection for transforming DNA. Our findings suggest both the existence of mechanisms that translate LiAc stress into lax control of rDNA copy number and that a disturbingly large number of strains generated using LiAc transformations may harbor copy number variants at the rDNA locus. Given the growing number of rDNA copy number repercussions being reported, LiAc-induced rDNA variation has the potential to be a significant passenger mutation with unanticipated influence in studies that examine genome replication, gene expression, DNA damage and responses, and chromatin regulation.

Inconsistencies in strains from the genome-wide deletion libraries are not uncommon and candidates identified from collections require validation by *de novo* strain construction, gene complementation, or genetic linkage experiments. In our studies, we found that two out of nine G418-resistant strains in the YKO collection had wild-type copies of their advertised gene deletions. While all of the isolates we tested from the Magic Marker collection had the correct barcodes for their advertised deletions, there was significant heterogeneity in rDNA size and ploidy across the isolates we examined. Our findings highlight the importance of validating strains from either collection when using them in screens or as sources of single gene deletion strains. In addition, care should be taken when using either collection to examine a phenotype that may be influenced by rDNA copy number since the rDNA sizes are unpredictable, heterogeneous within a strain, and may yield different results depending on the single colony chosen. Because of these problems, it is impractical to use either of these collections to screen for regulators of rDNA copy number. Difficulty in identifying true gene deletions that confer rDNA copy number changes is further compounded by the high frequency of rDNA variants that arise from strains generated by standard transformation protocols. These genome-wide deletion libraries most likely contain gene deletions that truly are responsible for rDNA copy number alterations, but our results suggested that a majority of the rDNA copy changes observed in YKO and Magic Marker collection strains do not result from the specific deleted gene. As we have found in our study, candidate mutations identified via genetic screens are meaningless without validation.

### No correlation found between lifespan and rDNA copy number

One of the primary goals of our study was to determine if a direct link exists between rDNA copy number and yeast replicative lifespan. We found no significant distinction between the distributions of rDNA copy number of the long-lived strains and the control strains. McCormick *et al.* (2015) comprehensively examined both the *MATa* and *MAT*α YKO deletion collection for their replicative lifespan analyses, including the same *MAT*α YKO strains we used in our rDNA copy number screen. If we assume that rDNA copy number is the same between different replicates of the YKO collections, our findings align with previous reports that wild-type yeast strains with different rDNA copy number have identical longevities (Michel *et al.* 2005; Saka *et al.* 2013). However, the rDNA copy number in a strain from one YKO set may not necessarily be the same in the same strain from another lab’s YKO set. Since the rDNA sizes of the YKO strains used in the longevity study were not examined, our results regarding the lack of correlation between lifespan and rDNA copy number may be inconclusive.

### Lithium acetate transformation as a potential source of rDNA size variation

It is unsettling that transformation using lithium acetate, a routine protocol used in yeast laboratories, has the potential to enrich for rDNA copy number variants. We found that exposure to lithium acetate generated more frequent and significant rDNA copy number variation than in untreated cells. Wild-type *S. cerevisiae* strains exhibit a low frequency of rDNA copy number fluctuation: 1% of wild-type colonies have a 40–60% reduction in rDNA array size, and colony sectoring experiments imply that rDNA reduction events happen within a single cell division (Michel *et al.* 2005). In comparison, approximately 10% of isolates taken through a mock transformation had significantly altered rDNA copy number, a value strikingly similar to the proportion of the gene knockouts from the YKO collection that Saka *et al.* (2016) reported as causing rDNA copy number change. Our results suggest, instead, that approximately 500 of the 4876 strains in the YKO genome-wide deletion collection strains could have random and genetically-unlinked rDNA changes. In keeping with this conclusion, we found no significant correlation between the deletion strains with altered rDNA copy number in our study compared to those reported by Saka *et al.* (2016) (Figure S2). While spheroplast transformation and electroporation both caused very little change in rDNA copy number, they are more cumbersome and/or inefficient transformation procedures (Kawai *et al.* 2010). We therefore expect that LiAc transformation will continue to be the standard protocol; however, individual researchers will need to consider carefully the potential for unanticipated phenotypes stemming from rDNA copy number changes.

We did not distinguish whether the increased frequency of rDNA copy number variants in LiAc transformants was due to selection for preexisting rDNA repeat variation or if the lithium acetate was inducing expansions/contraction of rDNA array. An interesting tangential finding was that these sudden changes to rDNA copy number were stably maintained instead of reverting to wild-type levels in contrast to previous studies of rDNA copy number perturbation (Kobayashi *et al.* 1998). The responsible pathways that distinguish the decision between rDNA copy number maintenance and recovery remain a mystery.

Why is lithium acetate inducing rDNA copy number changes? It is possible that rDNA copy number variation is a general cellular response to stress. Electron micrographs of *S. cerevisiae* cells treated with transformation solutions of 100 mM lithium acetate + PEG show a more porous cell wall than that of cells treated with PEG alone (Pham *et al.* 2011). Additionally, studies using lithium chloride have found that lithium toxicity can be seen in *S. cerevisiae* cells exposed to 100 mM LiCl (Demae *et al.* 2007; Ruiz *et al.* 2006), similar to the concentration of lithium in LiAc transformation protocols, and protein phosphatases have been documented to play key roles in lithium response. Loss-of-function mutants of the yeast calcineurin PP2B phosphatase Cna1/2, PP2C phosphatase Ptc1, and PP1 phosphatase Glc7 show increased cation sensitivity, while constitutively active forms of these phosphatases can confer tolerance of otherwise toxic lithium concentrations (Ferrer-Dalmau *et al.* 2010; Mendoza *et al.* 1996; Ruiz *et al.* 2006). In addition to its role in ion homeostasis, Glc7 plays an important part in regulating DNA replication initiation (Hiraga *et al.* 2014). The replication state of the cells has been previously shown to influence rDNA copy number (Ide *et al.* 2007) and, if competition for Glc7 exists between LiAc and DNA replication, the fluctuations we see in rDNA copy number may be reflecting transient effects of lithium in cells actively dividing during transformation.

To our knowledge, lithium-induced DNA copy number variation has not been previously reported in *S. cerevisiae*, but recent studies in other organisms report relevant observations that could warrant future investigation into the extracellular factors that influence rDNA maintenance. LiAc transformation-associated chromosome loss has been documented in *Candida albicans*. Aneuploid *C. albicans* have greater chromosome loss rates when taken through LiAc transformation using either heat shock or electroporation, though the authors only confirm that heat shock can increase chromosome loss (Bouchonville *et al.* 2009). Additionally, the modulation of rDNA copy number in response to environmental changes is not specific to yeast alone. A recent study has shown that *D. melanogaster* exhibits rDNA instability in response to dietary excess, presumably through transcription/replication conflicts at rDNA loci, and culminates in significant loss of chromosomal rDNA copies (∼20%) that is inherited by subsequent generations (Aldrich and Maggert 2015). If the mechanism behind LiAC-generated rDNA copy number variation stems from similar replication conflicts, it could suggest a potentially conserved mechanism of rDNA copy number regulation in response to environmental cues.

## Supplementary Material

Supplemental Material
