# Supplementary material for: rDNA Copy Number Variants Are Frequent Passenger Mutations in Saccharomyces cerevisiae Deletion Collections and de Novo Transformants
Source: G3 (Bethesda). 2016 Jul 22;6(9):2829–38. doi: 10.1534/g3.116.030296 (PMC5015940; doi:10.1534/g3.116.030296)
Supplement: Supplemental Material [file supp_6_9_2829__index.html]

rDNA Copy Number Variants Are Frequent Passenger Mutations in Saccharomyces cerevisiae Deletion Collections and de Novo Transformants — Supplemental Material 

# rDNA Copy Number Variants Are Frequent Passenger Mutations in *Saccharomyces cerevisiae* Deletion Collections and *de Novo* Transformants

## Supplemental Material for Kwan, *et al*, 2016

**Files in this Data Supplement:**

- Figure S1 - Verification of rDNA copy number estimates. (.tif, 1.13 MB)
- Figure S2 - rDNA copy number measurements from two independent screens of the YKO collection. (.tif, 1.17 MB)
- Figure S3 - rDNA copy number variation in the A364a laboratory strain BB14-3a (McCune *et al.,* 2008) after lithium acetate transformation. (.tif, 993 KB)
- Figure S4 - Chromosome XII size variation of clones taken through individual steps of the lithium acetate transformation protocol. (.tif, 11.42 MB)
- Figure S5 - rDNA copy number variation after transformation by electroporation or spheroplasting. (.tif, 2.24 MB)
- Table S1 - List of primers used in this study. (.xlsx, 11 KB)
- Table S2 - List of YKO strains examined and estimated rDNA copy number. (.xlsx, 28 KB)
- File S1 - This file contains the complete legends for all supplemental figures. (.docx, 119 KB)
