# Supplementary figures and images for: rDNA Copy Number Variants Are Frequent Passenger Mutations in Saccharomyces cerevisiae Deletion Collections and de Novo Transformants
Source: G3 (Bethesda). 2016 Jul 22;6(9):2829–38. doi: 10.1534/g3.116.030296 (PMC5015940; doi:10.1534/g3.116.030296)

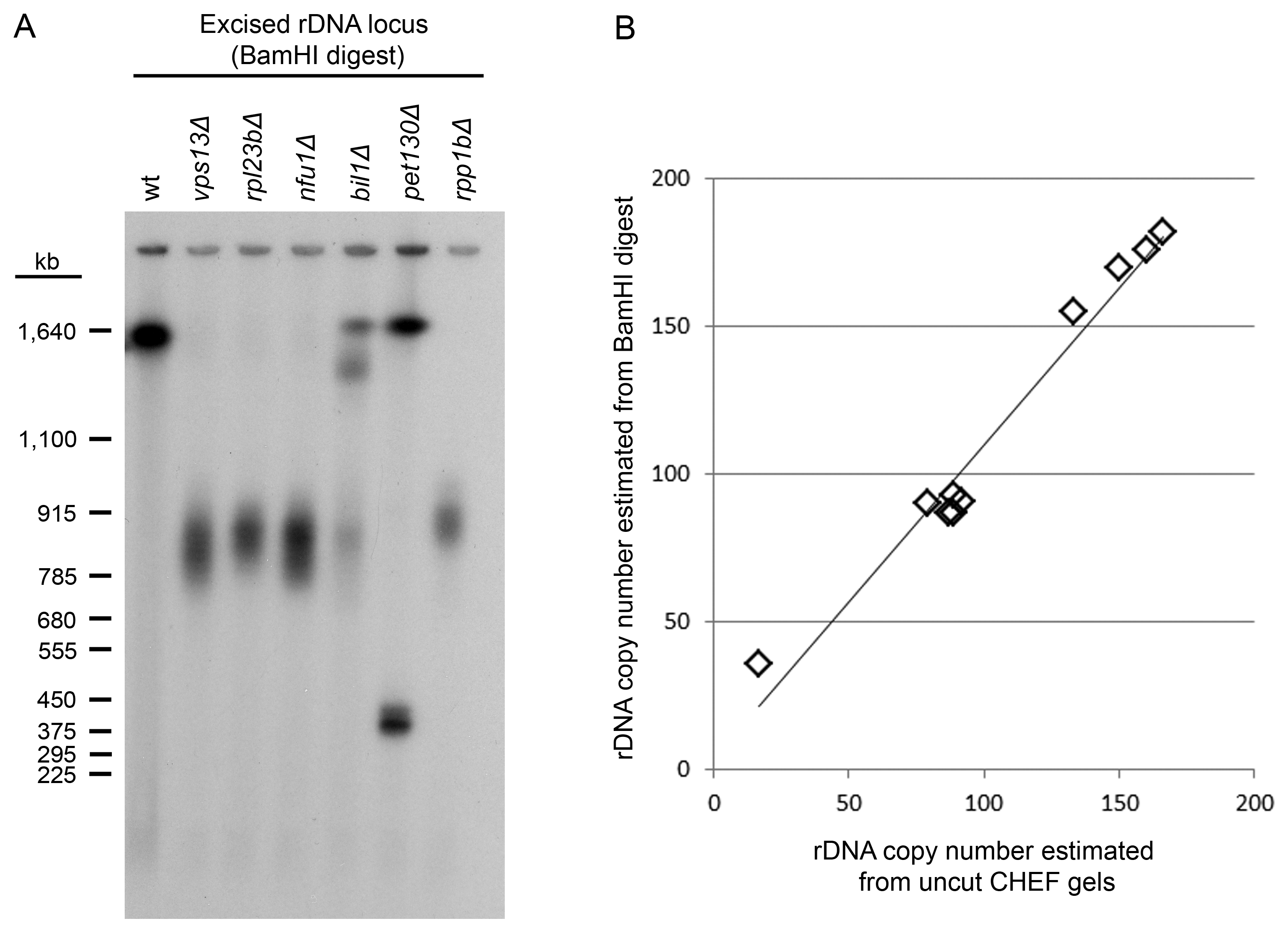

Supplement: Supplemental Material [file supp_g3.116.030296_FigureS1.tif]

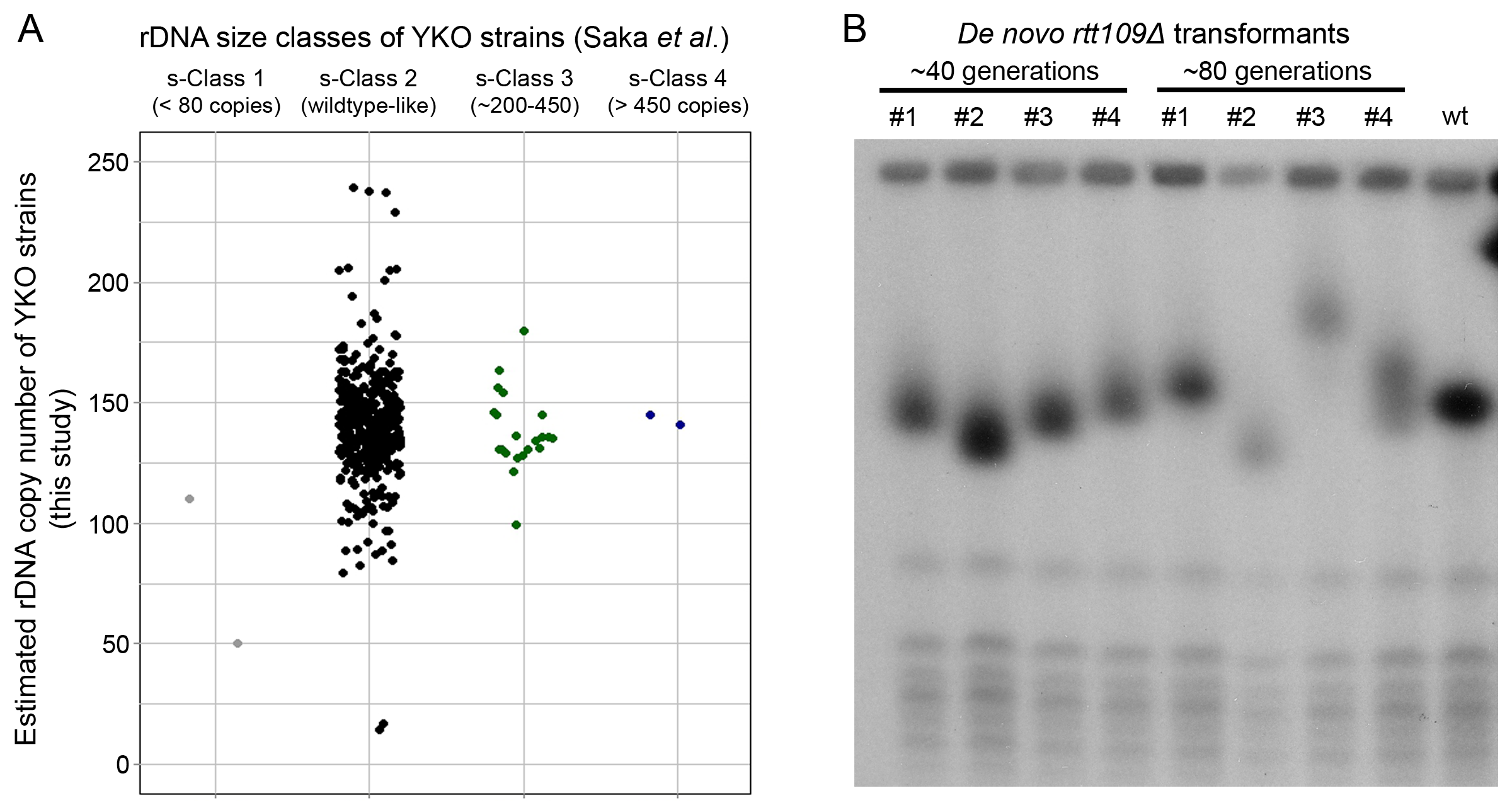

Supplement: Supplemental Material [file supp_g3.116.030296_FigureS2.tif]

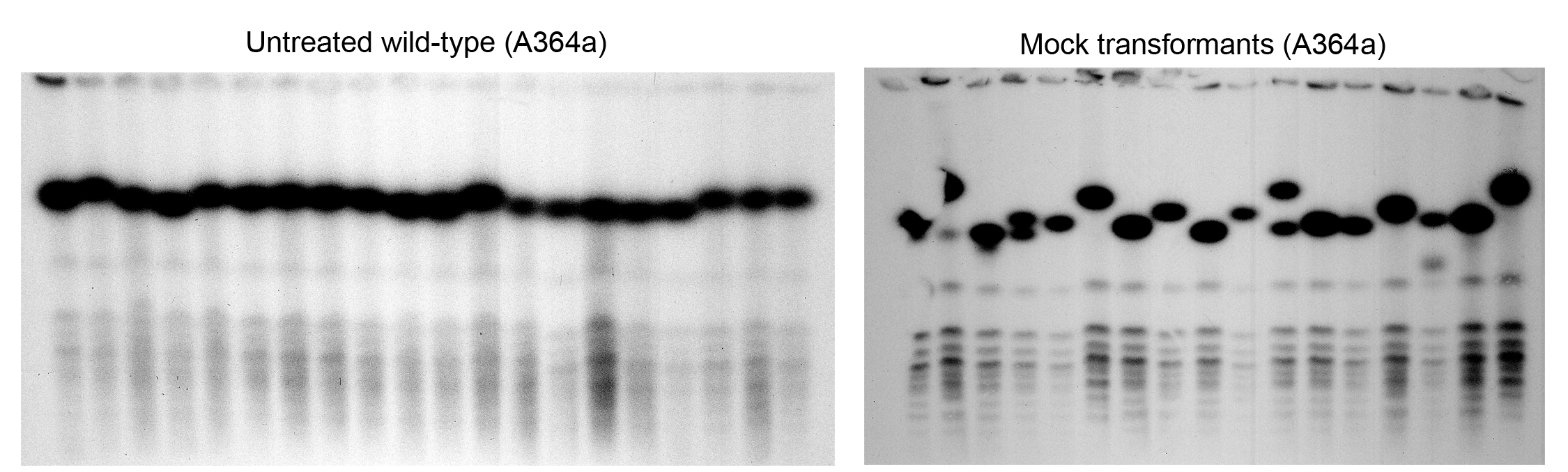

Supplement: Supplemental Material [file supp_g3.116.030296_FigureS3.tif]

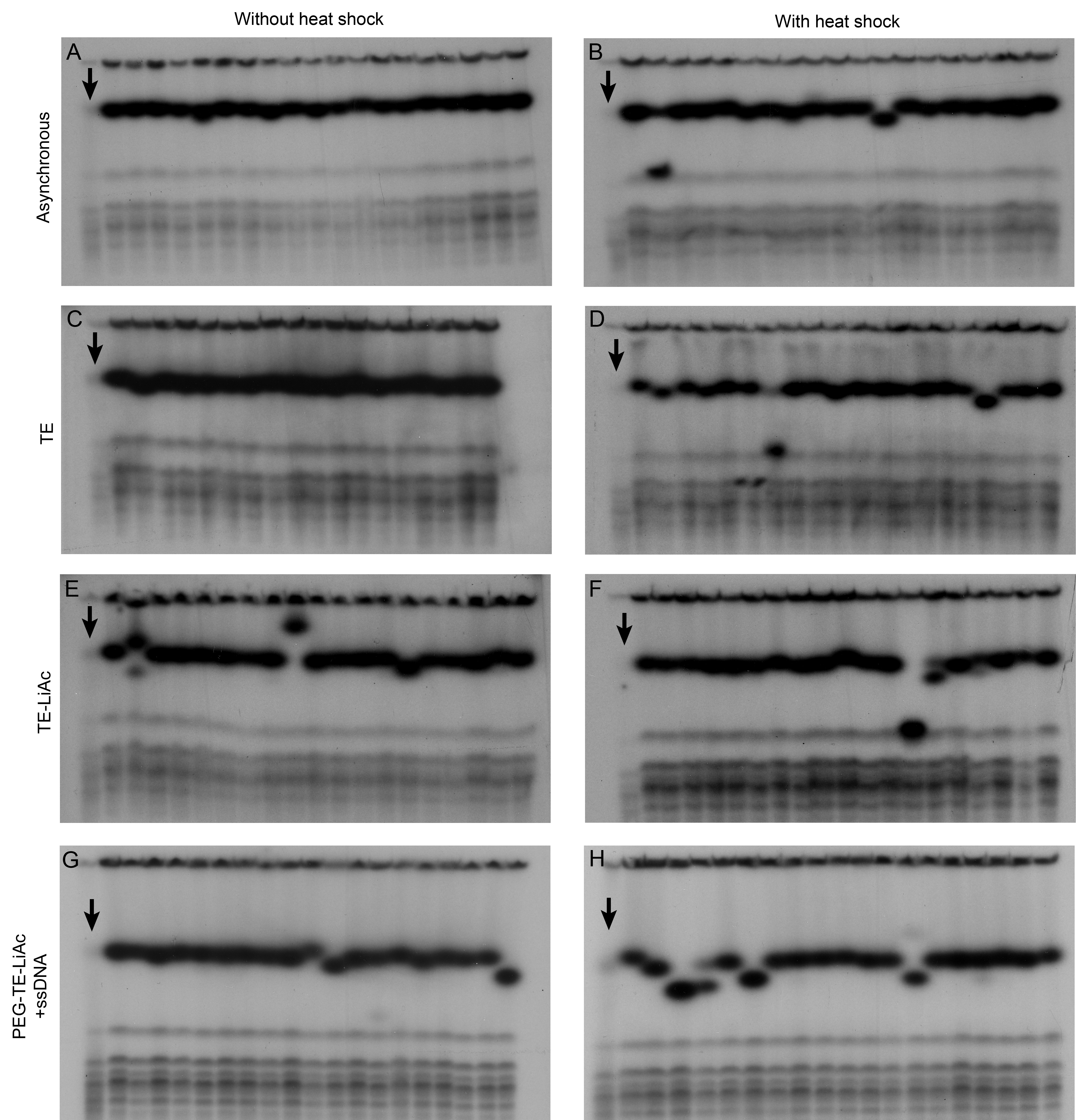

Supplement: Supplemental Material [file supp_g3.116.030296_FigureS4.tif]

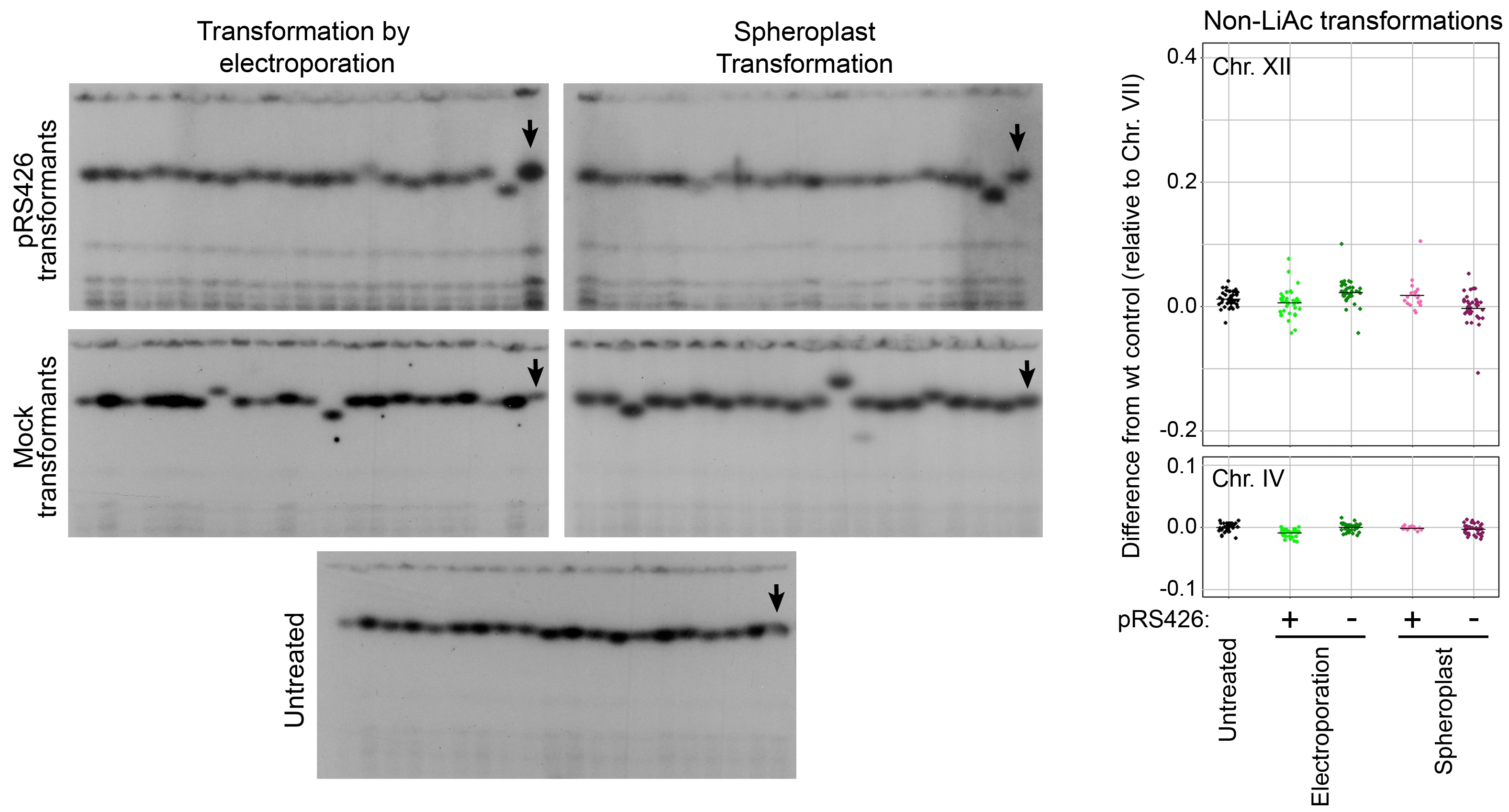

Supplement: Supplemental Material [file supp_g3.116.030296_FigureS5.tif]
