# Supplementary material for: rDNA Copy Number Variants Are Frequent Passenger Mutations in Saccharomyces cerevisiae Deletion Collections and de Novo Transformants
Source: G3 (Bethesda). 2016 Jul 22;6(9):2829–38. doi: 10.1534/g3.116.030296 (PMC5015940; doi:10.1534/g3.116.030296)
Supplement: Supplemental Material [file supp_g3.116.030296_FileS1.docx]

Figure S1: Verification of rDNA copy number estimates. Since the rDNA array contains no *Bam*HI sites, *Bam*HI digestion allows the measurement of the intact rDNA array away from its chromosome XII context (the rDNA *Bam*HI fragment contains 39.7 kb flanking sequence, Figure 1A). (A) *Bam*HI digested YKO samples examined using CHEF gel electrophoresis and hybridization to *RNH203,* which is within the *Bam*HI sites flanking the rDNA array*.* The multiple rDNA array bands in the YKO *bil1Δ* and *pet130Δ* strains indicate the presence of a mixed population of cells with different rDNA copy numbers (also visible in Figure 2). The size of each rDNA array was determined by comparing it to the locations of the chromosomes in the wild-type (uncut) sample visualized in the ethidium bromide stained CHEF gel. (B) Comparison of rDNA copy number estimates from uncut chromosome XII sizes (Figures 1 and 4) with the rDNA copy number determined from the size of the *Bam*HI-excised rDNA array (R² = 0.968).

Figure S2: rDNA copy number measurements from two independent screens of the YKO collection. Saka *et al.* (2016) categorized their YKO strains into four classes based on chromosome XII/rDNA size: 1 (< 80 rDNA copies), 2 (similar to wild-type copy number), 3 (200-450 rDNA copies), and 4 (> 450 rDNA copies). (A) Comparison of rDNA copy number measurements of the 434 YKO strains examined in this study with the YKO screen by Saka *et al.* (2016) (R = 0.039, *p* = 0.413). (B) CHEF gel analysis of chromosome XII size in four independent *de novo* *rtt109Δ* transformant strains generated from the *MATa* S288c strain BY4741. Each strain was examined after being passaged for 40 and 80 generations. rDNA copy number of the *de novo rtt109Δ* transformants did not stabilize at the greatly increased ~400 copies observed in the YKO screen performed by Ide *et al*. (2013).

Figure S3: rDNA copy number variation in the A364a laboratory strain BB14-3a (McCune *et al.,* 2008) after lithium acetate transformation. CHEF gel Southern blots probed for *RNH203,* a single copy gene on chromosome XII. Right panel: 20 clones from an untreated BB14-3a cell culture. Left panel: 17 BB14-3a clones from cells taken through lithium acetate transformation without selectable DNA.

Figure S4: Chromosome XII size variation of clones taken through individual steps of the lithium acetate transformation protocol. CHEF gel electrophoresis used to examine chromosome XII size of 38 clones (19 shown) from BY4741 cultures plated for single colonies as described below. (A) Asynchronous (without heat shock): untreated cells (plated directly from the culture); (B) asynchronous (with heat shock): culture incubated at 42°C for 30 minutes; (C) TE (without heat shock): cells incubated in TE at room temperature for 30 minutes; (D) TE (with heat shock): cells incubated in TE at 42°C for 30 minutes; (E) TE-LiAc (without heat shock): cells incubated in TE-LiAc at room temperature for 30 minutes; (F) TE-LiAc (with heat shock): cells incubated in TE-LiAc at 42°C for 30 minutes; (G) PEG-TE-LiAc + ssDNA (without heat shock): cells incubated in PEG-TE-LiAC + ssDNA (sheared and denatured herring testes DNA) at 30°C for 30 minutes; PEG-TE-LiAc + ssDNA (with heat shock); (H) cells incubated in PEG-TE-LiAC + ssDNA at 30°C for 30 minutes and then 42°C for 30 minutes. A common wild-type untreated control sample was included on each gel (arrows).

Figure S5: rDNA copy number variation after transformation by electroporation or spheroplasting. (A) Chromosome XII-probed CHEF gels of 19 independent colonies from electroporation and spheroplast transformation protocols without LiAc. Bottom panel: 19 untreated clones from the asynchronous culture used for both the electroporation and spheroplast transformations. A common untreated control sample was included on each gel (arrows). (B) Variation of chromosomes XII and IV in single colonies from electroporation or spheroplast transformation, with and without pRS426 plasmid DNA, was compared with untreated control colonies (Wilcoxon Rank-Sum: *p* > 0.2).
